# Targeting DNA Damage Repair for Immune Checkpoint Inhibition: Mechanisms and Potential Clinical Applications

**DOI:** 10.3389/fonc.2021.648687

**Published:** 2021-05-07

**Authors:** Wei Sun, Qing Zhang, Runkun Wang, Yang Li, Yue Sun, Lin Yang

**Affiliations:** ^1^ Department of Oncology, Tongji Hospital, Tongji Medical College, Huazhong University of Science and Technology, Wuhan, China; ^2^ Department of Ultrasonic Diagnosis, Peking Union Medical College Hospital, Chinese Academy of Medical Sciences and Peking Union Medical College, Beijing, China; ^3^ Department of Oncology, The First People’s hospital of Guangshui, Hubei, China

**Keywords:** immune checkpoint inhibitors, predictive biomarker for prognosis, target therapy, DNA damage repair, combined therapy

## Abstract

DNA damage repair (DDR) pathways play an essential role in maintaining genomic integrity. DDR dysfunction leads to accumulated DNA damage, predisposition to cancer, and high sensitivity to chemotherapy and radiotherapy. Recent studies have demonstrated that DDR status is associated with response to immune checkpoint inhibitors (ICIs). Among the DDR pathways, mismatch repair is one of the most recognized predictive biomarkers for ICIs. Furthermore, preclinical and early clinical studies suggest the rationale of combining agents targeting the DDR pathways, such as poly (ADP-ribose) polymerase (PARP) inhibitors, cyclin-dependent kinase 4/6 (CDK4/6) inhibitors, and ataxia telangiectasia and rad3-related (ATR) kinase inhibitors, with ICIs. In the present review, we describe the predictive role of DDR pathways in ICIs and summarize the advances in potential combination strategies of novel agents targeting DDR with ICIs for cancer treatment.

## Introduction

Immunotherapy, especially immune checkpoint inhibition (ICI), has reshaped the cancer treatment landscape and has become a standard therapy for multiple cancer types owing to its robust and durable anti-tumor response (1–3). However, the efficacy of immune checkpoint inhibitors (ICIs) varies widely, and only few cancer patients can benefit from ICIs. Currently, ICIs are expensive, and accurate predictive biomarkers for ICIs are lacking. Therefore, identifying patients who will benefit from ICIs and how to further improve the clinical outcome of ICIs represent the most significant challenge during the clinical application of immunotherapy.

DNA damage repair (DDR) pathways, which repair DNA damage caused by endogenous and exogenous factors, are essential for maintaining DNA fidelity in actively replicating cells. Consequently, a dysfunction of the DDR pathways induces genomic instability and tumor evolution, which is a hallmark of cancer. To date, accumulating preclinical and clinical evidence indicates that alterations in tumor DDR pathways are highly correlated with tumor susceptibility to ICIs (4, 5). Additionally, DDR machinery dysfunction has been demonstrated to elicit the host immune system’s activation, suggesting a potential treatment strategy for combining agents targeting DDR with ICIs (6–8).

In recent years, new agents targeting DDR pathways have been developed and explored extensively (9). Furthermore, there is an increasing number of ongoing clinical trials focusing on the combinational therapy of DDR targeted agents with ICIs. In this review, we address the predictive role of DDR pathways in ICIs and the attractive strategies of combining agents targeting DDR with ICIs for cancer treatment.

## Predictive Role of DDR in ICI

The established biomarkers for ICB include programmed death-ligand 1 (PD-L1) expression, tumor mutation burden (TMB), and mismatch repair (MMR) deficiency. However, neither is sufficient to precisely select beneficiaries for immunotherapy. For instance, the response rate of ICIs in high-TMB (≥20 mutations per Mb) cases is only 58%, whereas 20% of patients have intermediate and low TMB response to ICIs (10). Increased somatic copy number alteration (SCNA), which is positively associated with high TMB, has been demonstrated to be an immune suppression marker (11). Increased SCNA is also associated with poor clinical outcomes from anti-programmed death-1 (PD-1) or anti-cytotoxic T lymphocyte-associated antigen-4 (CTLA-4) blockade therapy (12, 13). Despite these findings, the threshold of PD-L1 expression and TMB for predicting response to ICI is still not definitive.

**Figure 1:**
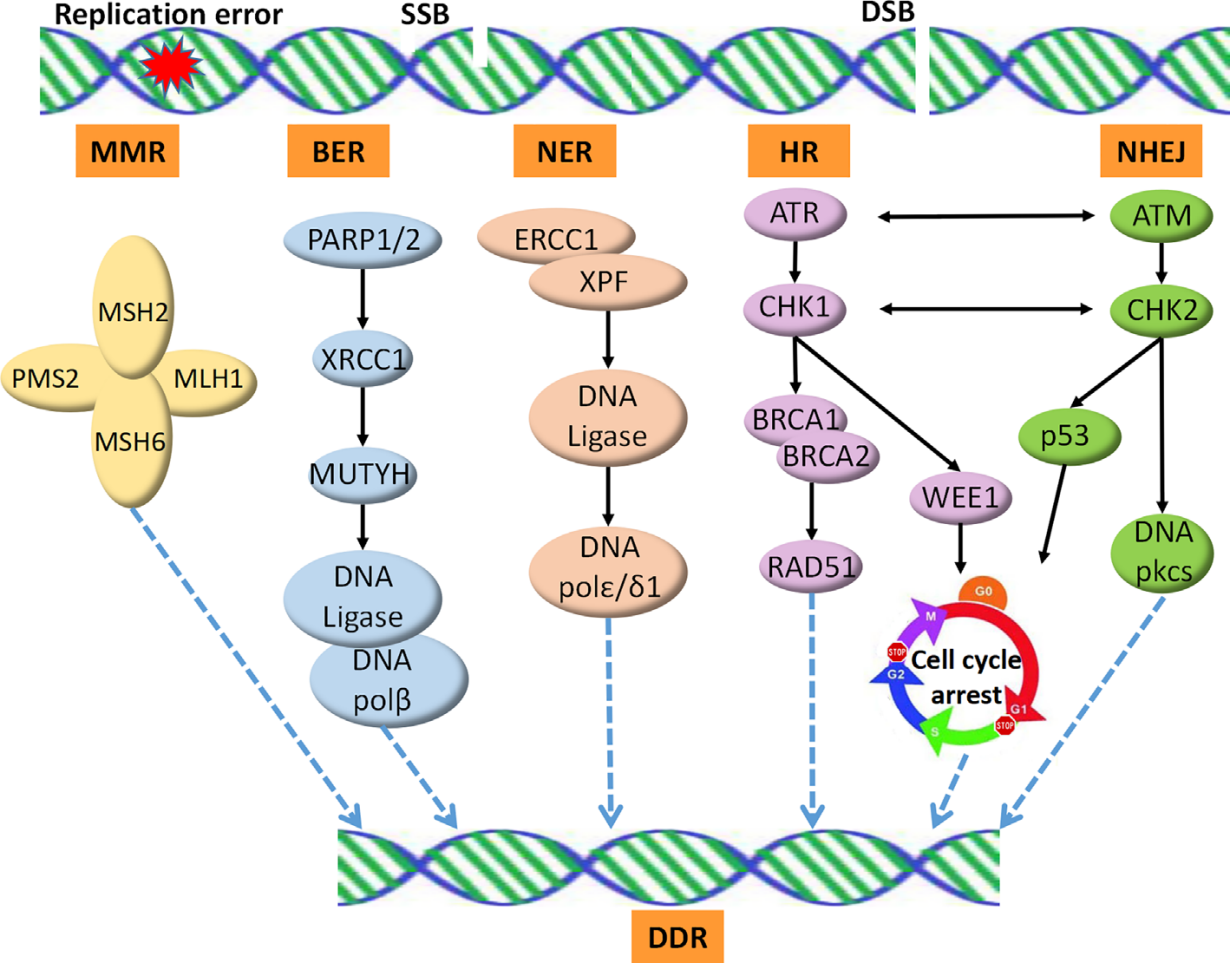
DNA damage repair pathways. SSB is repaired by the BER, NER, or MMR machineries. DSB is repaired by HRR, an accurate DNA repair pathway, or NHEJ, which is an error-prone pathway. SSB, single strand break; DSB, double strands break; MMR, mismatch repair; NER, nucleotide excision repair; BER, base excision repair; HR, homologous recombination repair; NHEJ, non-homologous end joining; DDR, DNA Damage Repair.

Recent studies have revealed that DDR profoundly impacts the interaction between the host immune system and cancer cells. Alterations in the DDR pathways could thus serve as reliable predictive biomarkers for the clinical application of ICIs (14). In total, over 450 proteins identified in the DDR pathways have been reported. These proteins are involved in five major functional pathways, including MMR, nucleotide excision repair (NER), base excision repair (BER), homologous recombination repair (HR), and non-homologous end joining (NHEJ) (**Figure 1**). MMR deficiency is one of the best-established predictive biomarkers of ICI therapy (15).

### MMR

MMR is essential for correcting errors in DNA replication. Deficient expression of any of the genes involved in the MMR pathway, including *MSH2*, *MSH6*, *MLH1*, and *PMS2*, leads to increased acquisition of mutations and either a gain or loss of nucleotides from microsatellite tracts, which exhibit a molecular feature of microsatellite instability-high (MSI-H) status (16). MSI-H status has been shown to contribute to Lynch syndrome and vulnerability to cancer (17). The most frequent MSI-H phenotype has been reported in endometrial and colorectal carcinomas, and more than 20 tumor types harbor the MSI-H phenotype at lower levels (16). The MSI-H phenotype presents high TMB and neoantigen burden, increased expression of PD-L1, and prominent immune cell infiltration, which are all associated with a remarkable response to ICIs (18, 19).

Owing to the favorable response of ICIs in cancers with MSI-H from clinical trials (4, 20), the anti-PD-1 drug pembrolizumab was approved for advanced MMR-deficient/MSI-H solid cancers, regardless of the tumor origin. Nivolumab, another anti-PD-1 drug, alone or in combination with ipilimumab, was also approved for advanced colorectal cancers with MMR deficiency.

### *BRCA1/2* and HR

HR, an important pathway for the precise repair of DNA double-strand breaks (DSBs), plays an essential role in maintaining genome stability. Germline mutations of the core members of HR, such as *BRCA1* and *BRCA2*, have been revealed to be vulnerable to hereditary breast and ovarian cancer syndromes (21). Recent studies have also demonstrated that HR deficiency is correlated with accumulated neoantigen load, high PD-L1 expression, increased levels of cytosolic DNA, and increased numbers of tumor-infiltrating lymphocytes (TILs) (22, 23). Therefore, the relationship between HR status and response to ICIs has been widely explored. Deleterious mutations in *BRCA2* were found to be enriched in anti-PD-1 responders with melanoma, which encouraged further research (24). Nonetheless, another study demonstrated that ovarian cancer, even with *BRCA1/2* mutations, had a modest response to anti–PD-1/PD-L1 (25). Therefore, the effect of *BRCA1/2* mutations and HR status on ICI response remains unclear. It seems that cancer types and molecular backgrounds have impact on the predictive role.

### DNA Polymerase Genes *ε* (*POLE*) and *δ* (*POLD1*)

*POLE* and *POLD1* encode exonuclease domains of major nuclear polymerases responsible for the NER function. The mutational prevalence of *POLE* is reported to be 2.79%, while that of *POLD1* is 1.37% across various cancer types (26). Studies have demonstrated that tumors with mutations in *POLD1* and *POLE* had remarkably high point mutation burden (27) and increased TIL numbers and PD-1/PD-L1 expression, suggesting deep and durable benefits from ICI therapy (28–30). A recent study investigated 47,721 patients with multiple cancer types and demonstrated a potential predictive role of *POLE/POLD1* mutations in beneficial outcomes for ICIs (26). Consistently, Junjun He et al. retrieved the genomic data of 21,074 Chinese patients with different cancer types and revealed the predictive value of *POLE/POLD1* mutations, especially those in the proofreading domain, in positive outcomes for ICIs. They further suggested that *POLE/POLD1* proofreading deficiency led to the MSI phenotype (31). Another study reported that *POLE* proofreading mutations elicited intra-tumoral immune responses in 295 patients with stage II colorectal cancer. These tumors with *POLE* proofreading mutations were more prone to be MSI-H and were assessed as extremely high TMB. Patients with *POLE* proofreading mutations had excellent outcomes, regardless of MSI status, suggesting that sequencing of all the exonuclease domains of *POLE* gene is recommended for patients with colorectal cancer (32). More prospective large-scale clinical trials are warranted to verify the predictive role of *POLE/POLD1* mutations for ICI, especially those in the proofreading domain. As the FDA has approved pembrolizumab for MSI in various cancer types, it would certainly be interesting to further explore the underlying relationship between *POLE/POLD1* mutations with MSI. Given patients with colorectal cancer and endometrial cancer harbor the most frequent *POLE* mutations (33), they should be addressed more on this issue.

### MutY Homolog (MUTYH)

MUTYH is involved in the BER pathway, which is best known for MUTYH-associated polyposis (MAP). MAP is an autosomal recessive condition that confers a 63% risk of colorectal cancer by age 60 (34). Preclinical studies have revealed that mutations in *MUTYH* represent distinct C>A transversion and increased lymphocytic infiltration in colorectal cancer, suggesting that tumors with *MUTYH* mutations may efficiently respond to ICIs (35, 36). However, the predictive role of *MUTYH* mutations in ICIs is still being explored.

### Genome of DDR

As more than 450 proteins have been identified in the DDR pathways, alterations in single gene contribute limitedly to the entire function of DDR. Therefore, the status of the genome of DDR or multiple key genes in DDR, would provide more comprehensive insights into the whole DDR capacity and achieve a more precise prediction of the response to ICIs. This hypothesis has been investigated for urothelial carcinoma (UC). Previous studies have revealed that the most common alterations in DDR genes in UC, including *ERCC2*, *ATM*, and others, were associated with increased mutation burden, high neoantigen load, and improved response rates to gemcitabine, cisplatin, and ipilimumab (anti-CTLA-4) (37–40). Teo et al. focused on 34 DDR genes, grouped into several major functional DDR pathways, in patients with metastatic UC treated with atezolizumab (anti-PD-L1) or nivolumab and reported that the presence of deleterious DDR alterations was associated with an improved response rate and survival (5). Interestingly, the DDR alterations are absent in patients with liver metastases (5), a well-known predictive factor for inadequate response to ICI therapy (41, 42), suggesting the molecular mechanism underlying the insufficient effect of ICI on liver metastases.

## DDR as a Potential Target for Combination With ICI

Although ICIs are approved for indications across different tumor types, the durable response rate for ICIs is only 10–20% (43). As a result, combinational strategies have been extensively explored to improve the clinical outcomes of ICI.

Several recent studies have shown significant survival benefits from the combinational therapy of ICIs with chemotherapy or radiotherapy (44, 45). Based on the findings of the KEYNOTE189 trial, pembrolizumab combined with chemotherapy has been approved as the first-line treatment for advanced non-small cell lung cancer (NSCLC) (44). Durvalumab has also been recognized as the standard maintenance treatment after concurrent chemoradiotherapy for locally advanced NSCLC, according to the results of the PACIFIC study (45, 46). Mechanically, chemotherapy and radiotherapy cause DNA damage, increase cytosolic DNA, and induce neoantigens, which trigger the host immune response (14). Nonetheless, cytotoxic chemotherapy and radiotherapy also kill host immune cells, which are required for an anti-tumor immune response. Moreover, chemotherapy and radiotherapy create subclonal mutations that are correlated with immune escape and inadequate response to ICIs (47). Therefore, basic and clinical research studies are increasingly focusing on ICIs combined with targeted therapy.

**Figure 2:**
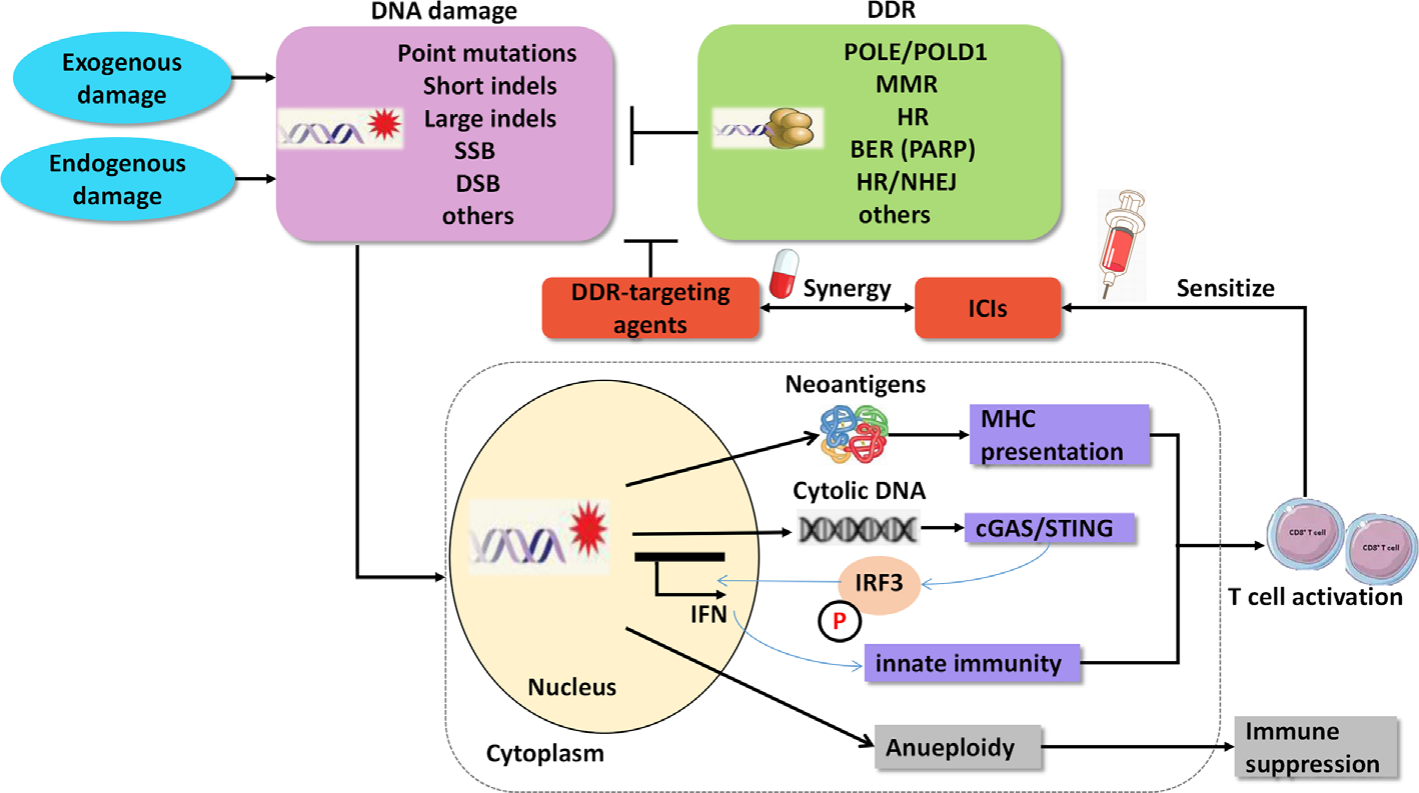
Agents targeting DNA damage repair synergize with immune checkpoint inhibitors. SSB, single strand break; DSB, double strands break; DDR, DNA damage repair; MMR, mismatch Repair; HR, homologous recombination; BER, base excision repair; PARP, poly ADP-ribose polymerase; NHEJ, non-homologous end Joining; cGAS, cGAMP synthase; STING, stimulator of interferon genes.

In addition to the predictive roles of the DDR pathways in ICI therapy, agents targeting DDR have important therapeutic implications in cancer, either as a monotherapy or in combination with other drugs, such as chemotherapy and ICIs. According to preclinical experiments, dysfunction of the DDR pathways reshapes the immune environment and contributes to the sensitization of ICIs (14) (**Figure 2**). The underlying mechanisms of the synergy are as follows: 1. DDR deficiency results in the accumulation of impaired DNA damage, including somatic mutations in exons, and yields mutant proteins called neoantigens. Neoantigens can elicit an anti-tumor immune response, including intratumoral CD8^+^ T-cell infiltration and cytolytic activity, and are associated with clinical response to ICIs (48–50); 2. Independent of neoantigens, accumulated damaged DNA, which transfers from the nucleus to the cytoplasm and is known as cytosolic DNA, can directly activate the stimulator of interferon genes *(STING)/TBK1/IRF3* pathway to induce type I interferon (IFN) response and trigger the innate immune response (51, 52). For instance, cancer cells with DDR dysfunction, such as mutations in *BRCA1/2* or *ATM*, show high levels of cytosolic DNA, which activates the STING pathway and innate immune response correlated with a durable response to ICIs (8, 53).

**Figure 3:**
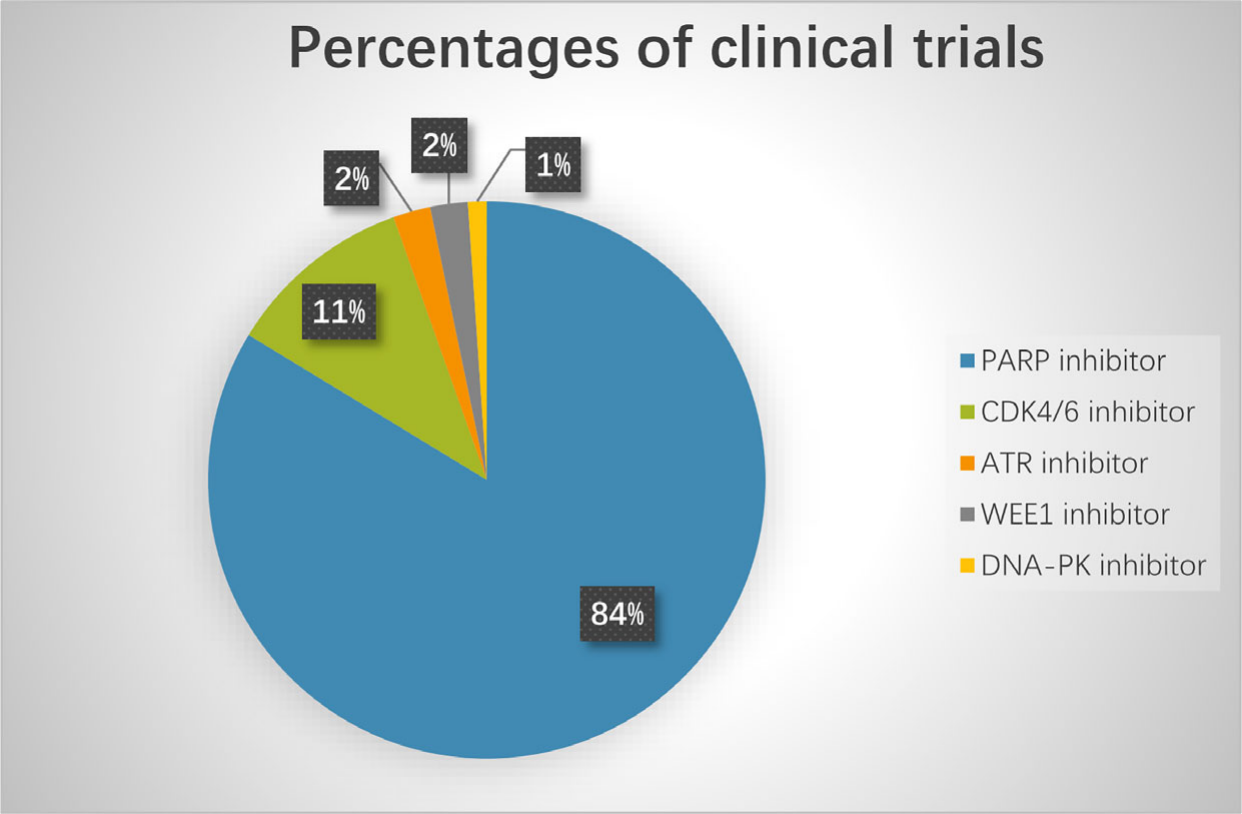
The percentage of clinical trials on combinational therapy with agents targeting DDR and immune checkpoint inhibitors. The total number of the clinical trials is 92.

Based on preclinical results, many clinical trials are currently exploring novel agents targeting DDR pathways combined with ICIs (**Figure 3**). Among these trials, PARP inhibitors combined with PD-1/PD-L1 inhibitors are the furthest in clinical development. Preliminary results have demonstrated that the combination of DDR-targeting agents with ICIs is a promising therapeutic strategy for cancer. These agents are summarized as follows:

### Poly (ADP-Ribose) Polymerase Inhibitors

Dysfunction of *BRCA1/2* or other genes in the HR pathway yields an HR-deficient phenotype and shows sensitivity to PARP inhibitors, which are classic examples of synthetic lethal therapy. Four PARP inhibitors, including olaparib, rucaparib, niraparib, and talazoparib, have been approved by the FDA to treat metastatic breast cancer, ovarian cancer, fallopian tube cancer, prostate cancer, and primary peritoneal cancer harboring deleterious germline mutations in *BRCA1/2* or with platinum-sensitivity properties (54). Nonetheless, PARP inhibitors only afford a progression-free survival (PFS) benefit of 2-4 months as a monotherapy. Even in patients with germline *BRCA* mutations, the overall survival (OS) benefit was not statistically significant (55). The incidence of intrinsic and acquired resistance to PARP inhibitors is high; therefore, studies assessing combinational strategies to improve efficacy are encouraged. PARP inhibitors in combination with ICIs are promising and have attracted extensive attention (**Table 1**).

**Table 1:** Clinical trials combining PARP inhibitors with immune checkpoint inhibitors.

DDR-targeting agents	Combined immune-checkpoint inhibitors	Trial registration number	Disease	Phase	Enrollment cases
olaparib	durvalumab	NCT03801369	Breast cancer	2	28
	durvalumab	NCT03167619		2	60
	durvalumab	NCT03544125		1	8
	durvalumab	NCT03594396		1/2	25
	durvalumab	NCT03737643	Ovarian cancer	3	1056
	durvalumab	NCT03699449		2	68
	Pembrolizumab	NCT03740165		3	1086
	durvalumab	NCT02953457	Ovarian/tubal or peritoneal cancer	2	39
	durvalumab ± cediranib	NCT02484404	Ovarian/breast/ lung/prostate/ colon/rectum cancer	1/2	384
	durvalumab	NCT03923270	SCLC	1	54
	Pembrolizumab	NCT03976323	Non-squamous NSCLC	3	792
	Pembrolizumab	NCT03976362	Squamous NSCLC	3	735
	Atezolizumab	NCT02849496	NSCLC	2	72
	durvalumab	NCT03810105	Prostate cancer	2	32
	Pembrolizumab	NCT02861573	Prostate cancer	1	400
	Pembrolizumab	NCT03834519	Prostate cancer	3	780
	durvalumab	NCT03534492	Urothelial/ bladder cancer	2	29
	durvalumab	NCT03459846	Urothelial cancer	2	150
	durvalumab	NCT02546661	Bladder cancer	1	156
	durvalumab	NCT03741426	Renal cancer	2	60
	durvalumab	NCT03579784	Gastric cancer	2	40
	durvalumab + cdk	NCT03784014	Soft tissue carcinoma	3	960
	durvalumab	NCT02882308	HNSCC	2	41
	durvalumab	NCT03772561	Solid tumor	1	40
	durvalumab	NCT03851614		2	90
	Durvalumab	NCT03842228		1	102
	durvalumab	NCT02734004 (MEDIOLA)		1/2	427
	durvalumab	NCT03991832		2	78
rucaparib	Nivolumab	NCT03522246	Ovarian cancer	3	1012
	Nivolumab	NCT03958045	SCLC	2	36
	Nivolumab	NCT03639935	Cholangiocarci-noma	2	35
	Nivolumab	NCT03572478	Prostate/endometrial cancer	1/2	60
	Nivolumab	NCT03824704	Solid tumor	2	139
niraparib	Atezolizumab	NCT03695380	Ovarian cancer	1	70
	Atezolizumab	NCT03598270	Ovarian/tubal/peritoneal cancer	3	414
	Pembrolizumab	NCT02657889 (KEYNOTE162/TOPACIO)	Ovarian cancer/Breast cancer	1/2	121
	Atezolizumab	NCT03869190	Urothelial cancer	1/2	305
	Nivolumab/ ipilimumab	NCT03404960	Pancreatic cancer	1/2	84
	Pembrolizumab	NCT03307785	Cancer	1	168
veliparib	Nivolumab	NCT03061188	Solid tumor/Lymphoma	1	50
	Nivolumab	NCT02944396	NSCLC	1	129
pamiparib	tislelizumab	NCT02660034	Solid tumor	1	230

PARP, Poly (ADP-ribose) polymerase; DDR, DNA damage repair; NSCLC, non-small cell lung cancer; SCLC, small cell lung cancer; HNSCC, head and neck squamous cell carcinoma.

In terms of the mechanism, PARP inhibitors inhibit DNA repair and aggravate DNA damage, which induces neoantigens and cytosolic DNA, activates the interferon pathway, triggers anti-tumor immunity, and converts immunologically “cold” tumors to “hot” tumors. Therefore, they could sensitize tumor cells to ICIs, especially under a BRCA-deficient background (55, 56). Despite these factors, PARP inhibitors upregulate the expression of PD-L1 through the inactivation of glycogen synthase kinase 3*α*/*β*, providing another rationale for the combination of PARP inhibitors with ICIs (56, 57). Moreover, accumulated evidence suggests that cancer stem cells (CSCs) are resistant to PARP inhibitors (58). However, CSCs exhibit a high expression of PD-L1 compared to non-CSCs in breast and colon cancer (59), and may be more sensitive to ICIs, which is one possible reason for the durable response and long survival benefit of immunotherapy. Consequently, combined ICIs may reverse the resistance of CSCs to PARP inhibitors, although direct evidence is still lacking (60).

#### Breast Cancer

In metastatic breast cancer, several trials have been designed to explore the combination of PARP inhibitors with ICIs, especially in triple-negative breast cancer (TNBC), which is enriched with *BRCA1* (70%) and *BRCA2* (20%) mutations, and is considered the most immunogenic subtype of breast cancer (61). The phase 2 trial, KEYNOTE-162/TOPACIO (NCT02657889), exploited the combination of niraparib with pembrolizumab in patients with advanced metastatic TNBC and recurrent ovarian cancer (62). In the TNBC cohort, among the 45 evaluable patients, three (6.67%) achieved complete response (CR), and 10 (22.22%) achieved partial response (PR). Patients with germline *BRCA* mutations had a higher objective response rate (ORR) of 66.67% (8/12) than others (62).

Additionally, patients with PD-L1-positive tumors responded better than those with PD-L1-negative tumors (33 *vs.* 15%). In the MEDIOLA trial (NCT02734004), a phase 1/2 open-label basket study, olaparib combined with durvalumab was investigated in solid tumors, including TNBC, ovarian cancer, small-cell lung cancer (SCLC), and gastric cancer. Patients received 300 mg olaparib as monotherapy daily for 4 weeks, and intravenous durvalumab was added at 1.5 g per 4 weeks (63, 64). The disease control rate (DCR) at 12 weeks was reported to be 80% in *HER-2* negative and *BRCA1/2* mutated metastatic breast cancer (63). The most commonly reported grade 3–4 adverse events were anemia, fatigue, neutropenia, and pancreatic enzyme elevation.

#### Ovarian Cancer

Higuchi et al. compared treatment with CTLA-4 or PD-1/PD-L1 antibodies alone or in combination to PARP inhibitors in *BRCA*-mutated ovarian tumors. They found that the CTLA-4 antibody, but not the PD-1/PD-L1 blockade, synergized therapeutically with PARP inhibitors, which led to immune-mediated tumor clearance and long-term survival (65). However, a phase 1 trial that evaluated olaparib combined with durvalumab in female cancers reported an ORR of 17% and a DCR of 83%. Notably, 11 of the 12 response tumors were negative for *BRCA* mutations. Inconsistently, in the KEYNOTE-162 study, a phase 2 trial exploiting the combination of niraparib with pembrolizumab, among the 60 recurrent ovarian cancer patients, the ORR was 25% and the DCR was 68%, whereas among the 11 patients with *BRCA* mutation, the ORR was 45% and the DCR was 73%. Thus, it is unclear whether *BRCA* mutation predicts a positive response to PARP inhibitors combined with ICIs, ultimately warranting further explanation.

A recent study using advanced genomic analyses and single-cell imaging reported that mutational signature 3 (a specific mutational signature reflecting defective HR) and positive immune score (a surrogate of interferon-primed exhausted CD8^+^ T-cells in the tumor microenvironment) determined response to niraparib plus pembrolizumab in patients with platinum-resistant ovarian cancer enrolled in the TOPACIO trial (66). Presence of one or both features associated with significantly prolonged PFS (HR = 0.32), while concurrent absence yielded no response. This study suggests that both biomarkers for PARP inhibitors and ICIs should be considered to predict the response to the combinational treatment.

Another focus of ovarian cancer is maintenance therapy using PARP inhibitors combined with ICIs. Three phase 3 randomized, double-blind, placebo-controlled multicenter studies have explored the effect of chemotherapy ± anti-PD-1/anti-PD-L1 followed by maintenance with anti-PD-L1 and olaparib/niraparib in newly diagnosed advanced ovarian cancer (NCT03737643/DUO-O), *BRCA* non-mutated advanced epithelial ovarian cancer (NCT03740165/KEYLYNK-001/ENGOT-ov43), and recurrent ovarian, tubal, or peritoneal cancer (NCT03598270). The results of the three phase 3 trials are worth expecting. Owing to the high tolerance of PARP inhibitors combined with ICIs, the maintenance strategy showed appealing preliminary results and potential for preventing tumor recurrence.

#### Lung Cancer

Despite the high TMB in small cell lung cancer (SCLC), most patients respond modestly to ICI therapy (67). Preclinical experiments suggested that olaparib activated the *STING/TBK1/IRF3* pathway in SCLC, but did not lead to T-cell recruitment or anti-tumor efficacy *in vivo*. Nonetheless, a recent study revealed that the addition of PD-L1 blockade could reverse these effects (7). The MEDIOLA study also included a cohort of patients with SCLC (67). In this cohort, patients received durvalumab 1,500 mg every 4 weeks and olaparib 300 mg twice daily. Among the 19 evaluable patients, two patients (10.5%) achieved PR or CR, including a patient with EGFR-transformed SCLC, and four patients (21.1%) had clinical control with confirmed responses or prolonged stable disease (≥8 months). However, this effect does not meet the preset bar, as the ORR of 10.5% failed to reject the null hypothesis that the ORR should be not less than 35%. The treatment-related adverse effects included anemia (80%), lymphopenia (60%), and leukopenia (50%). Notably, all tumors with an inflamed phenotype (CD8-positive T cells directly contacting the tumor) responded to the combination. The results suggest that the tumor immune phenotype plays a predictive role in response to the combination of PARP inhibitors with ICIs in SCLC, which should be confirmed in larger cohorts.

In NSCLC, two phase 3 studies are being conducted to explore the effect of pembrolizumab combined with olaparib in maintenance therapy following pembrolizumab combined with chemotherapy as the first-line treatment (NCT03976323 for non-squamous, NCT03976362 and MK-7339-008/KEYLYNK-008 for squamous NSCLC). A phase 2 study using durvalumab combined with olaparib in patients with NSCLC who did not respond to anti-PD-1/PD-L1 therapy is also in progress (NCT03334617). The results of these studies will be worth exploring. Furthermore, predictive biomarkers are warranted to select patients to improve the clinical outcomes of combinational therapy.

#### Prostate Cancer

In prostate cancer, several clinical trials are currently being conducted. The KEYNOTE-365, a phase 1b/2 umbrella study, evaluated pembrolizumab with olaparib in heavily pretreated metastatic castration-resistant prostate cancer (mCRPC). Among the 39 evaluable patients, the PSA response rate (reduction of ≥50% in serum PSA levels) was 13%, while the radiological ORR was 7%, and the DCR was 29%. None of the tumors harbored mutations in the HR genes. A phase 2 clinical trial (NCT02484404) demonstrated that olaparib combined with durvalumab was effective for mCRPC, and the toxicity was acceptable (68). The PSA response rate was 53%, while the radiological ORR was 24%, and the PFS rate at 1 year was 51%. Interestingly, patients with DDR gene alterations achieved a PFS rate at 1 year of 83% compared to 36% in those without DDR gene alterations (p = 0.031). These results suggest that DDR gene alterations may be used as predictive markers for guiding combinational therapy in patients with prostate cancer.

#### Other Cancers

In the MEDIOLA study, 40 patients with gastric cancer who relapsed after platinum-based chemotherapy received durvalumab and olaparib (64). The DCR was only 26% at 12 weeks, and the ORR was 10%. Due to the poor activity of olaparib as a single agent in this setting, all responses occurred after the addition of durvalumab, which suggests that the combination strategy deserves additional large-scale prospective investigation for recurrent gastric cancer.

The most recently published multicenter, open-label, phase 1a/b trial (NCT02660034) from Australia, investigated the safety and anti-tumor effects of pamiparib, an oral PARP1/2 inhibitor, combined with tislelizumab, a humanized anti-PD-1 monoclonal antibody, in patients with advanced solid tumors (60). Ten patients (10/49, 20%) achieved objective responses, including two CR and eight PR. Twenty-three patients (23/49, 47%) had immune-related adverse events, of whom nine (39%) had asymptomatic grade 3-4 hepatic injury, which was reversible with corticosteroid treatment.

The combination of PARP inhibitors with ICIs has also been widely explored in head and neck squamous cell carcinoma (HNSCC), soft tissue sarcoma, renal cancer, other solid cancers, and lymphoma (**Table 1**). More randomized trials are needed to confirm whether the efficacy of the combination is superior to PARP inhibitor or ICIs monotherapy.

### Inhibitors of Cyclin-Dependent Kinase 4/6

Inhibitors of CDK4/6, including palbociclib, ribociclib, and abemaciclib, have been approved to treat patients with hormone receptor-positive, HER2-negative metastatic breast cancer (69–71). As outlined earlier, CDK4/6 inhibitors inhibit the proliferation of cancer cells by suppressing retinoblastoma (RB) phosphorylation and maintaining the repressive effect of RB on the E2F family, thereby reducing the transcription of pro-proliferative proteins and inhibiting cell cycle progression. Several studies have revealed that CDK4/6 inhibitors can inhibit DDR and enhance the radiosensitivity of NSCLC, glioblastoma, and HNSCC cells (72–74). Importantly, CDK4/6 inhibition was demonstrated to repress HR by inhibiting critical HR factors, such as Rad51 (75).

CDK4/6 inhibitors not only induce cell cycle arrest, but also enhance tumor immunogenicity, which provides the rationale for combination with ICIs (76). Several mechanisms underlying this synergy have been identified. The mechanisms include: CDK4/6 inhibitors reduce the proliferation of immunosuppressive T-reg cells (76), increase tumor antigen presentation in breast cancer cells, stimulate type III IFN production (76), and promote the infiltration and activation of T-cells. However, the proliferation of T-cells is suppressed (77). The inhibition of CDK4/6 increases the expression level of the PD-L1 protein by suppressing ubiquitination-mediated PD-L1 degradation (78). Additionally, the synaptonemal complex protein 3 (SCP3)-cyclin D1-CDK4/6 axis is activated during the immunoediting process, which drives tumor cells to generate acquired resistance to immunotherapy. However, CDK4/6 inhibitors could reverse the multi-aggressive phenotypes of SCP3 in immune-refractory cancer and lead to a long-term response to ICIs (79). Finally, the resistance program of ICI in melanoma, which is associated with T cell exclusion and immune evasion, could be repressed by CDK4/6 inhibitors (80).

Findings from a preclinical study showed that CDK4/6 inhibitors combined with anti-PD-L1 therapy led to tumor regression in animal cancer models (81). Similarly, Teo et al. demonstrated that the combination of CDK4/6 inhibitors with ICIs (targeting PD-1 and CTLA-4) and PI3Kα inhibitors induced complete and durable regression (>1 year) in established xenograft mouse models of human TNBC (82).

In a clinical trial, the first phase 1/2 study was designed to explore the potential of abemaciclib (LY2835219) plus pembrolizumab in patients with HR-positive metastatic breast cancer (NCT02779751) (83). A total of 28 patients were enrolled. The preliminary results showed that four patients (14%) achieved an objective response at 24 weeks, which was higher than the response rate (11%) reported in the MONARCH 1 study with abemaciclib monotherapy (70). Currently, a multicenter phase 2 study is being conducted to evaluate the combination of letrozole, palbociclib, and pembrolizumab in postmenopausal women with HR-positive advanced breast cancer (NCT02778685) (84). All three FDA-approved inhibitors of CDK4/6 are now in clinical trials in combination with ICIs to treat cancers, such as breast cancer, HNSCC, NSCLC, and liver cancer (**Table 2**).

**Table 2:** Clinical trials combining CDK4/6 inhibitors with immune checkpoint inhibitors.

DDR targeting agents	Combined immune checkpoint inhibitors	Trial registration number	Disease	Phase	Enrollment
abemaciclib	nivolumab	NCT03655444	HNSCC	1/2	32
	pembrolizumab	NCT02079636	NSCLC	1	150
	pembrolizumab	NCT02779751	NSCLC/ breast cancer	1	100
	pembrolizumab	NCT03997448	GEA	2	34
	nivolumab	NCT03781960	Liver cancer		
palbociclib	pembrolizumab	NCT02778685	Breast cancer	2	22
	Avelumab (+Fulvestrant)	NCT03147287		2	220
	Avelumab (+Tamoxifen)	NCT03573648		2	40
trilaciclib	atezolizumab	NCT03041311	SCLC	2	105
Dinacidib	Pembrolizumab	NCT01676753	Breast cancer	1	32

CDK, cyclin-dependent kinase; DDR, DNA damage repair; NSCLC, non-small cell lung cancer; SCLC, small cell lung cancer; HNSCC, head and neck squamous cell carcinoma; GEA, gastro-esophageal adenocarcinoma.

### Inhibitors of Ataxia Telangiectasia and Rad3-Related (ATR) Kinase

ATR kinase is a known master regulator of DDR. Therefore, ATR is an attractive therapeutic target for cancer treatment, especially in combination with DNA-damaging agents. A previous study showed that ATR inhibition decreased the expression of PD-L1 by destabilizing PD-L1 in a proteasome-dependent manner to attenuate PD-1/PD-L1 interaction and sensitized cancer cells to T cell killing, which provided a rationale for the combination therapy of ATR inhibitors with other types of ICIs, such as anti-CTLA-4 or anti-TIM-3 (85).

Clinical trials exploring the ATR inhibitor, AZD6738, combined with durvalumab in patients with advanced solid tumors (NCT02264678, phase 1/2) and NSCLC who have progressed on anti-PD-1/PD-L1 containing therapy (NCT03334617, phase 2) are ongoing. The initial data showed an acceptable toxicity profile and promising preliminary anti-tumor activity. A patient with HNSCC and another patient with NSCLC also achieved PR (86). Another study recruited participants to evaluate the efficacy and safety of avelumab in combination with an ATR inhibitor (M6620) and carboplatin in PARP inhibitor-resistant, recurrent, platinum-sensitive ovarian, primary peritoneal, or fallopian tube cancer.

### Inhibitors of WEE1

WEE1 is a protein kinase that activates the G2/M cell cycle checkpoint by inhibiting cyclin-dependent kinase 1 and 2 (CDK1/2) and provides time for DDR (87). Inhibition of WEE1 impairs G2/M cell cycle arrest, hampers DDR, and increases replication stress. To date, AZD1775 (MK1775, Adavosertib) is the only WEE1 inhibitor used in clinical trials. Currently, two clinical trials are exploring the combination of adavosertib with durvalumab. One is NCT02546661 (BISCAY), a phase 1b randomized multi-drug biomarker­directed study in patients with metastatic muscle-invasive bladder cancer. In this clinical trial, patients with any HR deficiency will receive durvalumab ± olaparib, and patients with *CDKN2A* or *RB1* deficiency and/or amplifications of *CCNE1, MYC, MYCL*, or *MYCN* will receive durvalumab ± adavosertib (88). The other trial is NCT02617277, a phase 1 trial to assess the safety and pharmacokinetics of adavosertib plus durvalumab in patients with advanced solid tumors (89). In this trial, 54 patients with colorectal, lung, and breast cancer were enrolled. The overall DCR of the combination was 36%, and the dose-limiting toxicities were fatigue, nausea, and diarrhea. The promising results of this phase 1 clinical trial called for additional phase 2 study, and the schedule of adavosertib 150 mg twice per day on days 15-17 and 22-24 combined with durvalumab 1,500 mg on day 1 of a 4-week cycle was recommended for phase 2 study. The exploration of predictive biomarkers in the BISCAY study will help to select suitable patients for this combinational strategy.

### Inhibitors of Checkpoint Kinase 1 (CHK1)

CHK1 plays a crucial role in DDR and genome stability. CHK1 inhibition has been explored as a potential anti-tumor therapy. Recently, the inhibitor of CHK1, prexasertib (LY2606368), was demonstrated to remarkably activate the *STING/TBK1/IRF3* innate immune pathway and increase the expression of PD-L1, which contributes to the infiltration and activation of cytotoxic T lymphocytes and significantly potentiates the efficacy of ICIs in SCLC *in vivo* (7). Another preclinical study demonstrated that SAR737, an oral CHK1 inhibitor, combined with low-dose gemcitabine, enhanced the effect of PD-L1 blockade in SCLC by modulating the immune microenvironment (90). These findings suggest that the combination of CHK1 inhibitors and ICIs deserves further evaluation in clinical trials, especially for patients with SCLC.

Currently, there are no well-established predictive markers for CHK1 inhibitors. Sen et al. identified MYC as a biomarker of prexasertib through proteomic analysis and suggested that CHK1 inhibitors might be especially effective in SLCL with *MYC* amplification or MYC protein overexpression (91). The predictive marker of CHK1 inhibitors combined with ICIs is being explored in ongoing clinical trials. However, further investigation of the combination’s safety is needed, as preliminary results demonstrated that neutropenia was the most frequent and severe adverse event, which can be harmful, although manageable (92).

### Inhibitors of ATM

ATM is an apical kinase of the DDR pathway, which makes it an attractive anti-tumor therapeutic target. Two ATM inhibitors, M3541, and AZD0156 are currently in phase 1 trials for the treatment of solid tumors. A recent study reported that the inhibition of ATM increased IFN signaling and sensitized pancreatic cancer to ICI therapy (93). We recently reported that the inhibition of the ATM/CHK2 pathway could activate the innate immunity of ARID1A-deficient cancers and enhance the anti-tumor effect of PD-L1 antibody (94). The promising effect of the preclinical studies demonstrated that the combination of an ATM inhibitor with ICIs is urgent to be investigated in clinical trials.

### Inhibitors of DNA-PK

DNA-PK plays a crucial role in the NHEJ pathway of DDR. Currently, three inhibitors of DNA-PK, namely M9831 (VX­984), nedisertib (M3814, MSC2490484A), and CC­115, are being evaluated in phase 1/2 trials. Recent studies have reported that human DNA-PK activates a STING-independent DNA sensing pathway to drive a robust and broad immune response (95). For the combined treatment, a clinical trial, NCT03724890, is now being carried out to determine a safe and tolerable DNA-PK inhibitor (nedisertib) dose combined with ICIs (avelumab) ± radiotherapy for participants with selected advanced solid tumors.

## Conclusions and Remaining Challenges

In conclusion, emerging evidence supports that alterations in the DDR pathways play potential predictive roles for ICIs. The combination of agents targeting DDR with ICIs has resulted in appealing anti-tumor effects in preclinical and clinical studies. These advances may contribute to a major step forward in cancer treatment. However, there are many questions still to be answered.

Although the FDA has approved MMR deficiency for the application of PD-1 blockade, the impact of DDR alterations on the response to ICIs is largely unknown. First, the underlying relationship between DDR gene alterations and other known biomarkers of ICIs, such as TMB, should be subject to intense investigation. Although DDR deficiency usually leads to a high TMB, it is not always the same. TMB refers to the overall quantity of tumor gene mutations, while a specific DDR deficiency may represent the quality of the gene alterations. It seems that only a small amount of tumor-specific neoantigens aroused from DDR alterations is highly immunogenic, which may explain why patients with low TMB still respond to ICIs. It is crucial to identify these neoantigens or the related DDR gene alterations, which will ultimately help to select patients for immunotherapies. Second, standardized and practical methods to assess DDR defects, such as HR deficiency (96), should be established.

For the combination strategy, developing predictive biomarkers to identify patients who will respond to agents targeting DDR combined with ICIs is essential (9). Although most ongoing clinical trials have been conducted with unselected patients, several clinical trials have been designed to explore the possible predictive markers for combinational therapy, including alterations in genes such as *ARID1A, ATM, ATRX, BRCA1, BRCA2, CDK12, CHEK1, CHEK2, CCNE1, MYC, MRE11, MSH2, PARP1, PIK3CA, POLD1, PPP2R2A, PTEN, RAD51B, XRCC2*, and MMR status (NCT03842228, NCT02546661). However, individual predictive biomarker for DDR-targeted agents or ICIs was unable to predict the response to the combinational therapy (66). The success of mutational signature 3 and immune score in selection of patients with ovarian cancer who would benefit from niraparib/pembrolizumab (66), suggests that the predictive biomarkers should be cooperated in future clinical trials of combinational therapy. Furthermore, the optimal combination timing and sequences, which should stimulate the anti-tumor immune response and improve anti-tumor efficacy with minimal toxicity, are still unclear. Various schedules of combinations have been utilized in multiple clinical trials. However, it is unlikely to find a unified pattern for all combination regimens. In fact, it seems to vary widely across tumor settings and genetic backgrounds. For example, in clinical trials, PARP inhibitors and CKD4/6 inhibitors were administered concurrently, before or after ICIs, in various situations. Briefly, concurrent administration of PARP inhibitors or CKD4/6 inhibitors with ICIs was carried out in most trials. However, when PARP inhibitors were used as maintenance therapy (for ovarian cancer, NCT03737643, NCT03740165 and NCT03598270; for NSCLC, NCT02944396), they were used following ICIs, while in some other clinical trials, PARP inhibitors and CDK4/6 inhibitors were used before ICIs (NCT03579784, NCT03842228 and NCT03781960).

Collectively, it is challenging to define the optimal predictive biomarker, clinical setting, and combining schedule. To answer these questions, it is essential to understand how tumor intrinsic genetic alterations affect anti-tumor immunology and how the drugs synergize with each other. In light of the recent promising preclinical and early clinical findings in this field, additional basic and clinical studies are warranted in the future.

## Author Contributions

All authors contributed to the conception and design of the review. WS wrote the first draft of the manuscript. QZ, RW, YL, and YS revised the draft of the manuscript. QZ and RW added and revised all the figures and tables. LY corrected different versions and finalized the manuscript. All authors contributed to the article and approved the submitted version.

## Conflict of Interest

The authors declare that the research was conducted in the absence of any commercial or financial relationships that could be construed as a potential conflict of interest.
